# Supplementary material for: Changes in the Physicochemical Properties of Chia (Salvia hispanica L.) Seeds during Solid-State and Submerged Fermentation and Their Influence on Wheat Bread Quality and Sensory Profile
Source: Foods. 2023 May 23;12(11):2093. doi: 10.3390/foods12112093 (PMC10252298; doi:10.3390/foods12112093)
Supplement: Supplementary file 1 [file foods-12-02093-s001.zip › ed_Supplementary File S4_Method for hardness and acrylamide determination_v1.pdf]

Analysis of bread hardness

Bread hardness was determined with Texture Analyser TA.XT2 (StableMicro Systems Ltd., Godalming, UK) as the energy required for the sample deformation. Bread slices of 2 cm thickness were compressed to 10% of their original height at a crosshead speed of 10 mm/s. The resulting peak energy of compression was reported as hardness.

Analysis of Acrylamide Concentration in Bread

The acrylamide concentration was determined according to the method of Zhang et al. [39] with modifications, which are described below. Two grams of sample were weighed in 50 mL centrifuge tube and diluted with 20 mL of distilled/deionized water. Tube was briefly vortexed (ZX3 Advanced VELP, Italy) to mix the contents of tube for 10 min. The tube was centrifuged at 4,000 rpm for 10 min with a centrifuge (Hermle Z 306, Germany). The 10 mL of the aqueous layer solution in 15 mL centrifuge tubes was cleaned with 100 µl Carrez I (85 mM $K_4[Fe(CN)_6] \times 3 H_2O$) and 100 µl Carrez II (250 mM $ZnSO_4 \times 7 H_2O$) solutions. The tubes were centrifuged at 4,000 rpm for 10 min. Acrylamide standard solution (30,4 µg/L). 15.2 mg of acrylamide analytical standard (99.8% purity) was weighed and dissolved in a 1000 mL volumetric flask and diluted with deionized water.

The obtained solution was diluted by pouring 2 mL of the obtained acrylamide solution into a 1000 mL measuring flask and diluted with deionized water. Three millilitres of the sample supernatant (or standard solution) was derivatized in a glass tube by adding 1.5 g of potassium bromide (KBr), 1 mL of potassium bromate solution (0.1 M, $KBrO_3$) and 0.3 mL of sulfuric acid solution (50 %, H_2SO_4). The resulting mixture was mixed in a shaker and kept for 2 h in a refrigerator (~4°C).

The derivative was neutralized by adding 250 µL of sodium thiosulphate solution (1 M, $Na_2S_2O_3 \times 5H_2O$) until the orange colour disappears. About 1.5 g of sodium chloride (NaCl) was added to the derivatization mixture and the mixture was extracted with ethyl acetate ($CH_3COOC_2H_5$) (2 × 5 mL). The collected ethyl acetate was concentrated with a concentration system (Christ CT 02-50, Osterode, Germany) at a temperature of 40°C and reduced pressure. The solvent was evaporated and dissolved in 0.5 mL of ethyl acetate (for the standard, in a volume of 3 mL). The 100 mg of anhydrous sodium sulphate (Na_2SO_4), 20 µL of triethylamine [$(C_2H_5)3N$] (20 µL of triethylamine in 0.5 mL of a concentrated derivatization solution) was added to the solution in a 15 mL centrifuge tube, mixed and centrifuged for 10 minutes (4000 rpm). The supernatant was analysed by GC–ECD. A gas chromatograph (GC) (Shimadzu GC-17A, Japan) was equipped with an electron capture detector (ECD) and an integrator to measure peak areas, and a thermostatted column. Capillary column such as Rxi-5Sil MS (Restek, Germany): length 30 m; inner diameter 0.25 mm-ϕ; stationary phase film thickness 0.25 µm-ϕ. Working conditions: injection volume 1 µL; column temperature gradient 70°C (hold 1 min), 3°C/min to 140 (hold 0.5 min), 15°C/min to 280 (hold 4 min). Mobile phase was nitrogen 18.0 cm/sec flow-rate, split 3.0; injector temperature 250°C, detector temperature 260°C, detector current 2 nA.
