# Supplementary material for: Changes in the Physicochemical Properties of Chia (Salvia hispanica L.) Seeds during Solid-State and Submerged Fermentation and Their Influence on Wheat Bread Quality and Sensory Profile
Source: Foods. 2023 May 23;12(11):2093. doi: 10.3390/foods12112093 (PMC10252298; doi:10.3390/foods12112093)
Supplement: Supplementary file 1 [file foods-12-02093-s001.zip › ed_Supplementary File S5_Method for volatile compounds_v1.pdf]

Analysis of Volatile Compound (VC) Profile in Bread Samples

Bread was left at room temperature for 12 h to cool down and then was analysed. The volatile compounds (VC) of samples were analysed by gas chromatography-mass spectrometry (GC-MS). A solid-phase microextraction (SPME) device with Stableflex™ fibre coated with a 50 μm PDMS-DVB-Carboxen™ layer (Supelco, USA) was used for analysis. For headspace extraction of bread samples, 2 g of sample, 10 mL of 1M phosphate buffer (pH = 3) and 50 μl of internal standard solution (0.939 mg/ml of valeric acid) were transferred to the 20 mL extraction vial, mixed, sealed with a polytetrafluoroethylene septum, and thermostatted at 60°C for 30 min before exposing the fibre in the headspace. For headspace extraction of bread samples, 2 g of sample were transferred to the 20 mL extraction vial, mixed, sealed with a polytetrafluoroethylene septum, and thermostatted at 60°C for 30 min before exposing the fibre in the headspace. The fibre was exposed to the headspace of the vial for 10 min and desorbed in an injector liner for 2 min (splitless injection mode). Prepared samples were analysed with a GCMS-QP2010 (Shimadzu, Japan) gas chromatograph and mass spectrometer. The following conditions were used for analysis: injector temperature 250°C, ion source temperature 220°C and interface temperature 260°C. Helium was used as a carrier gas at 0.65 mL/min flowrate. For separation of VC, a low polarity Rxi®-5MS column (Restek, USA) (length 30 m, coating thickness 0.25 μm-ϕ, inner diameter of 0.25 mm-ϕ) was used. The temperature gradient was programmed from starting at 40°C (3 min hold) to 220°C (5°C/min) up to 310°C (15°/min) (6 min hold). The VC were identified according to mass spectrum libraries (NIST11, NIST11S, FFNSC2). NIST library data contains spectrum and retention index of the analyte. By using retention index and the spectrum matching, more false positive results can be removed. Minimal spectrum matching criteria was 85 percent. For identification purposes, alkane mix (C8-C20) were analysed to obtain the retention indexes of unknown compounds.
