# Supplementary material for: Changes in the Physicochemical Properties of Chia (Salvia hispanica L.) Seeds during Solid-State and Submerged Fermentation and Their Influence on Wheat Bread Quality and Sensory Profile
Source: Foods. 2023 May 23;12(11):2093. doi: 10.3390/foods12112093 (PMC10252298; doi:10.3390/foods12112093)
Supplement: Supplementary file 1 [file foods-12-02093-s001.zip › ed_Supplementary File S6_sensory analysis of bread_v1.pdf]

Sensory characteristics and overall acceptability of breads

Sensory characteristics and overall acceptability of breads were carried out according to the ISO 11136:2014 and ISO 8586:2012 by 30 trained panellists (20 females and 10 males) aged between 20 and 36 years. Panellists were usually consumers of bread. Three 60-min sessions were conducted for the training on the selected terms and the tasting procedure. According to quantitative descriptive sensory analysis, the sensory profile of samples was analysed. The intensity of colour, odour, flavour, acidity, bitterness, porosity, brittleness, springiness, hardness and moisture of the bread were assessed using a 10-point scale, where 0 and 10 indicate the lowest and the highest intensity, respectively. Overall acceptability was evaluated using a 10-point Likert scale ranging from 10 (extremely like) to 0 (extremely dislike). The evaluation was carried out at the sensory laboratory with individual booths and following standard sensory practices. The bread samples were baked and cooled down for 12 h before the sensory evaluation. All samples were coded and served randomly for evaluation. During sensory evaluation, panellists were instructed to drink water or rinse their mouths to clear the palate after each evaluation.
