# Supplementary material for: Changes in the Physicochemical Properties of Chia (Salvia hispanica L.) Seeds during Solid-State and Submerged Fermentation and Their Influence on Wheat Bread Quality and Sensory Profile
Source: Foods. 2023 May 23;12(11):2093. doi: 10.3390/foods12112093 (PMC10252298; doi:10.3390/foods12112093)
Supplement: Supplementary file 1 [file foods-12-02093-s001.zip › ed_Supplementary File S7_Volatile compounds_v1.pdf]

Table S1. Volatile compounds of the bread samples (% from the total volatile compounds).

RT, min	Volatile compounds	Bread samples									
		B_{C}	B_{NF10}	B_{NF20}	B_{NF30}	B_{SMF10}	B_{SMF20}	B_{SMF30}	B_{SSF10}	B_{SSF20}	B_{SSF30}
4.735	Ethanol	14.0± 1.10 f	9.92± 0.58 d	16.5± 0.15 g	2.93± 0.28 a	2.89± 0.25 a	8.20± 0.69 c	10.7± 0.09 e	2.86± 0.27 a	5.79± 0.49 b	8.70± 0.63 c
11.628	3-Methyl-1-butanol	25.3± 1.81 g	17.5± 0.18 f	24.6± 0.22 g	6.83± 0.59 a	7.18± 0.56 a	14.1± 0.13 c	15.0± 0.14 d	7.84± 0.76 a	11.0± 0.10 b	15.6± 0.14 e
13.602	2-Methylpyrazine	nd	1.43± 0.13 a	nd	3.87± 0.35 c	5.70± 0.52 d	2.23± 0.19 b	1.99± 0.18 b	3.34± 0.31 c	2.15± 0.19 b	nd
15.392	2-Ethylpyrazine	1.57± 1.12 a,b,c	2.63± 0.24 c	1.90± 0.17 b	4.23± 0.41 d	5.49± 0.53 e	2.74± 0.25 c	2.40± 0.22 c	3.74± 0.32 d	2.82± 0.25 c	1.34± 0.12 a
15.748	1-Hexanol	5.28± 0.45 b	4.99± 0.38 b	7.06± 0.56 c	5.23± 0.49 b	1.19± 0.11 a	9.03± 0.08 d	8.73± 0.08 d	9.20± 0.75 d	6.73± 0.58 c	9.02± 0.79 d
16.941	Tetradecane	5.07± 0.49 e	3.98± 0.37 d	2.76± 0.25 b,c	3.70± 0.36 c,d	5.17± 0.48 e	3.11± 0.27 b,c	2.60± 0.25 b	4.62± 0.42 d,e	3.24± 0.29 c	1.88± 0.17 a
18.374	2-ethyl-3,5-dimethylpyrazine	nd	1.36± 0.14 c	0.675± 0.059 a	3.74± 0.35 e	3.43± 0.29 e	1.53± 0.14 c	1.61± 0.15 c	2.19± 0.20 d	1.56± 0.14 c	0.824± 0.075 b
18.611	Acetic acid	3.26± 0.29 a,b	3.96± 0.35 b	4.84± 0.42 c	5.23± 0.51 c	3.35± 0.31 b	3.93± 0.29 b	3.94± 0.32 b	3.01± 0.28 a,b	2.77± 0.26 a	3.69± 0.34 b
19.063	3-Furaldehyde	1.31± 0.07 b	1.47± 0.13 b	nd	6.33± 0.62 d	5.65± 0.52 d	4.82± 0.41 c,d	4.56± 0.42 c	5.93± 0.57 d	5.14± 0.48 c,d	0.656± 0.061 a
19.388	2-Propyl-1-pentanol	1.06± 0.09 c	1.03± 0.11 c	0.709± 0.069 b	0.419± 0.32 a,b	0.506± 0.48 a,b,c	0.603± 0.059 b	0.340± 0.029 a	0.614± 0.054 b	0.491± 0.41 a,b	0.585± 0.059 b
20.127	1-(2-furanyl)-ethanone	nd	1.30± 0.12 b	nd	2.12± 0.20 d	2.32± 0.22 d	1.08± 0.09 a	1.27± 0.11 a,b	2.06± 0.21 d	1.67± 0.15 c	nd
20.655	Benzaldehyde	2.29± 0.21 a,b	3.60± 0.30 c	2.87± 0.27 b	3.03± 0.29 b,c	2.22± 0.19 a	3.06± 0.27 b,c	3.16± 0.29 b,c	2.27± 0.19 a	2.73± 0.24 b	2.89± 0.26 b
21.401	2-methylpropanoic acid	3.81± 0.32 d	2.31± 0.21 c	2.76± 0.29 c	1.24± 0.12 a	1.41± 0.13 a,b	2.31± 0.23 c	1.64± 0.15 b	1.89± 0.15 b	2.76± 0.23 c	2.63± 0.25 c
21.891	5-methyl-2-furancarboxaldehyde	nd	nd	nd	2.26± 0.21 b	2.24± 0.19 b	1.35± 0.12 a	1.40± 0.11 a	2.02± 0.18 b	2.01± 0.18 b	nd
22.087	Caryophyllene	nd	2.72± 0.26 d,e	0.666± 0.061 a	3.14± 0.30 e	2.27± 0.21 d	1.20± 0.11 b	1.81± 0.17 c	2.29± 0.21 d	2.40± 0.22 d	2.49± 0.23 d
23.499	Benzeneacetaldehyde	nd	10.2± 0.15 e	3.00± 0.29 a	14.9± 0.13 h	11.3± 0.09 g	4.06± 0.39 b	8.62± 0.79 d	9.39± 0.85 d,e	6.95± 0.52 c	11.0± 0.10 f
23.728	3-Furanmethanol	2.44± 0.23 a	3.47± 0.35 b	nd	7.91± 0.78 e	13.5± 0.12 f	4.76± 0.45 c	4.03± 0.41 b,c	7.14± 0.69 d,e	6.19± 0.49 d	2.12± 0.19 a
23.831	2-Methyl-butanoic acid	7.49± 0.52 d	4.39± 0.41 b	5.62± 0.51 c	3.17± 0.30 a	2.97± 0.25 a	5.46± 0.52 c	4.15± 0.40 b	4.29± 0.35 b	9.56± 0.79 e	7.55± 0.25 d
27.778	Hexanoic acid	10.1± 0.11 d	9.51± 0.09 c	12.5± 0.13 g	8.45± 0.79 b	6.29± 0.56 a	15.6± 0.14 j	11.9± 0.12 f	13.7± 0.12 h	11.0± 0.10 e	14.5± 0.11 i
29.372	Phenylethyl alcohol	11.3± 0.12 g	8.88± 0.11 d	9.09± 0.08 e	3.32± 0.21 a	4.44± 0.42 b	6.01± 0.38 c	5.59± 0.51 c	4.88± 0.41 b,c	8.22± 0.68 d	10.3± 0.09 f
30.026	Heptanoic acid	1.34± 0.14 c	0.904± 0.038 b	1.15± 0.09 c	0.626± 0.059 a	0.803± 0.079 b	1.15± 0.11 c	0.964± 0.089 b,c	1.19± 0.11 c	0.845± 0.63 a,b,c	0.934± 0.056 b
30.650	Maltol	nd	1.38± 0.14 c	nd	3.93± 0.33 e	6.38± 0.058 f	1.33± 0.12 c	0.907± 0.075 b	2.45± 0.22 d	1.58± 0.13	0.760± 0.038 a
32.167	Octanoic acid	3.64± 0.29 e	2.42± 0.22 c	3.00± 0.23 d	1.45± 0.13 a	1.70± 0.16 a,b	1.77± 0.15 b	2.02± 0.19 b,c	1.97± 0.17 b	1.72± 0.16 a,b	2.03± 0.18 b,c
35.083	4-Vinyl-guaiacol	0.816± 0.073 c	0.625± 0.059 b	0.278± 0.025 a	1.90± 0.09 f	1.58± 0.13 e	0.588± 0.056 b	0.605± 0.049 b	1.09± 0.10 d	0.746± 0.51 a,b,c,d	0.546± 0.049 b

RT – retention time; C – control bread, without chia seeds; B – bread; 10, 20, 30 – amount of the chia added (% from the flour content); NF – non-fermented; SMF – submerged fermentation; SSF – solid-state fermentation; total volatile compounds – refers to the identified compounds only. Data expressed as mean values (n = 3) ± standard error (SE).

a–j Mean values within a line with different letters are significantly different ($p \leq 0.05$).
