# Supplementary material for: Changes in the Physicochemical Properties of Chia (Salvia hispanica L.) Seeds during Solid-State and Submerged Fermentation and Their Influence on Wheat Bread Quality and Sensory Profile
Source: Foods. 2023 May 23;12(11):2093. doi: 10.3390/foods12112093 (PMC10252298; doi:10.3390/foods12112093)
Supplement: Supplementary file 1 [file foods-12-02093-s001.zip › Supplementary File S1_Method of LAB counts_v1.pdf]

LAB Viable Count Determination in Chia Seed Samples

For the evaluation of LAB viable counts, 10 g of sample was homogenised with 90 mL of aqueous saline (9 g/L NaCl solution). Serial dilutions of 10^{-4} to 10^{-8} with the same saline solution were prepared for inoculation. Sterile MRS (Man, Rogosa, Sharpe) agar (CM0361, Oxoid) of 5 mm thickness was used for bacterial growth in Petri dishes. The dishes were separately seeded with the sample suspension using surface sowing and were incubated under anaerobic conditions at 30 °C for 72 h. All results were expressed in $\log_{10}$ colony-forming units (CFU)/mL, considering the dilution factor and the amount of sample, and the average values and standard deviations (STDVs) of three analytical determinations were calculated. In microbial viable counts, the logarithmic transformation is required for stabilization of variance and normalization of residuals.
