# Supplementary material for: Changes in the Physicochemical Properties of Chia (Salvia hispanica L.) Seeds during Solid-State and Submerged Fermentation and Their Influence on Wheat Bread Quality and Sensory Profile
Source: Foods. 2023 May 23;12(11):2093. doi: 10.3390/foods12112093 (PMC10252298; doi:10.3390/foods12112093)
Supplement: Supplementary file 1 [file foods-12-02093-s001.zip › Supplementary File S2_Method for biogenic amines_v1.pdf]

Determination of Biogenic Amine Content in Chia Seed Samples

The extraction and determination of BA in samples followed the procedures developed by Ben-Gigirey et al. [33] with some modifications. The following BA were analysed: tryptamine (TRP), phenylethylamine (PHE), putrescine (PUT), cadaverine (CAV), histamine (HIS), tyramine (TYR), spermidine (SPRMD) and spermine (SPRM) . The standard BA solutions were prepared by dissolving known amounts of each BA (including internal standard—1.7-diamino-heptane) in 20 mL of deionised water. Briefly, 5 g of sample was extracted with 10 mL of perchloric acid (0.4 mol/L) twice. The derivatization of sample extracts and standards was performed using a dansyl chloride solution in acetonitrile (10 mg/mL) as a reagent. A Varian ProStar high-performance liquid chromatography (HPLC) system (Varian Corp., Palo Alto, CA, USA) equipped with a ProStar 325 ultra-violet/visible (UV/VIS) Detector and Galaxy software (Agilent, Santa Clara, CA, USA) was used for analysis. A Discovery® HS C18 column (150 × 4.6 mm-ϕ, 5 µm-ϕ; SupelcoTM Analytical, Bellefonte, PA, USA) was used to separate BA. Ammonium acetate (0.1 mol/L) and acetonitrile were used as the mobile phases at a flow-rate of 0.8 mL/min. The sample volume injected was 20 µL and the amines were monitored at 254 nm. The BA were identified based on their relative retention times (RRT) in comparison to their corresponding standards.
