# Supplementary material for: Changes in the Physicochemical Properties of Chia (Salvia hispanica L.) Seeds during Solid-State and Submerged Fermentation and Their Influence on Wheat Bread Quality and Sensory Profile
Source: Foods. 2023 May 23;12(11):2093. doi: 10.3390/foods12112093 (PMC10252298; doi:10.3390/foods12112093)
Supplement: Supplementary file 1 [file foods-12-02093-s001.zip › Supplementary File S3_Method for fatty acids determination_v1.pdf]

Analysis of Fatty Acid (FA) Profile of Chia Seed Samples

The extraction of lipids for fatty acids (FA) analysis was performed with chloroform/methanol (2:1, *v*/*v*), and FA methyl esters (FAME) were prepared according to Pérez-Palacios et al. [34] with some modifications. The fatty acid composition of samples was identified using a gas chromatograph GC-2010 Plus (Shimadzu Europa GmbH, Duisburg, Germany) equipped with Mass Spectrometer GCMS-QP2010 (Shimadzu Europa GmbH, Duisburg, Germany). Separation was carried out on a Stabilwax-MS column (30 m length, internal diameter 0.25 mm-ϕ and df 0.25 μm-ϕ) (Restek Corporation, Bellefonte, PA, USA). Oven temperature program started at 50 °C, then increased at a rate of 8 °C/min to 220 °C, held for 1 min at 220 °C, increased again at a rate of 20 °C/min to 240 °C and, finally, held throughout 10 min. The injector temperature was 240 °C, interface −240 °C, and ion source 240 °C. The carrier gas was helium at a flow-rate of 0.91 mL/min. The individual FAME peaks were identified by comparing their relative retention times (RRT) with FAME standards (Merck & Co., Inc., Kenilworth, NJ, USA).
